# Fenugreek extract as an inducer of cellular death via autophagy in human T lymphoma Jurkat cells

**DOI:** 10.1186/1472-6882-12-202

**Published:** 2012-10-30

**Authors:** Nasser M Al-Daghri, Majed S Alokail, Khalid M Alkharfy, Abdul Khader Mohammed, Sherif H Abd-Alrahman, Sobhy M Yakout, Osama E Amer, Soundararajan Krishnaswamy

**Affiliations:** 1 Biomarkers Research Program, Department of Biochemistry, College of Science King Saud University, PO Box 2455, Riyadh 11451, Saudi Arabia; 2 Prince Mutaib Chair for Osteoporosis Research, College of Science, King Saud University, Riyadh 11451, Saudi Arabia; 3 Department of Clinical Pharmacy, College of Pharmacy, King Saud University, Riyadh 11451, Saudi Arabia

**Keywords:** Jurkat cells, Fenugreek, Autophagy, LC3, Autophagic vacuoles, Chemoprevention, Anticancer

## Abstract

**Background:**

Drugs used both in classical chemotherapy and the more recent targeted therapy do not have cancer cell specificity and, hence, cause severe systemic side effects. Tumors also develop resistance to such drugs due to heterogeneity of cell types and clonal selection. Several traditional dietary ingredients from plants, on the other hand, have been shown to act on multiple targets/pathways, and may overcome drug resistance. The dietary agents are safe and readily available. However, application of plant components for cancer treatment/prevention requires better understanding of anticancer functions and elucidation of their mechanisms of action. The current study focuses on the anticancer properties of fenugreek, a herb with proven anti-diabetic, antitumor and immune-stimulating functions.

**Method:**

Jurkat cells were incubated with 30 to 1500 μg/mL concentrations of 50% ethanolic extract of dry fenugreek seeds and were followed for changes in viability (trypan blue assay), morphology (microscopic examination) and autophagic marker LC3 transcript level (RT-PCR).

**Results:**

Incubation of Jurkat cells with fenugreek extract at concentrations ranging from 30 to 1500 μg/mL for up to 3 days resulted in cell death in a dose- and time-dependent manner. Jurkat cell death was preceded by the appearance of multiple large vacuoles, which coincided with transcriptional up-regulation of LC3. GC-MS analysis of fenugreek extract indicated the presence of several compounds with anticancer properties, including gingerol (4.82%), cedrene (2.91%), zingerone (16.5%), vanillin (1.52%) and eugenol (1.25%).

**Conclusions:**

Distinct morphological changes involving appearance of large vacuoles, membrane disintegration and increased expression of LC3 transcripts indicated that fenugreek extract induced autophagy and autophagy-associated death of Jurkat cells. In addition to the already known apoptotic activation, induction of autophagy may be an additional mechanism underlying the anticancer properties of fenugreek. This is the first report showing fenugreek as an inducer of autophagy in human cells and further work is needed to define the various intermediates of the autophagic pathway.

## Background

Currently used drugs in cancer treatment cause severe side effects, since the targeted pathways also occur in rapidly dividing normal cells [1]. Newly emerging targeted drugs rely on inhibiting an aberrant tumor-promoting pathway and the heterogeneity of cell types enable cancers develop drug resistance [2]. In addition, genetic instability enables malignant cells to evade inhibitor drugs by secondary mutations in target genes and other genetic changes [2]. As a consequence, progress has been lacking in treating the majority of cancers.

Plant constituents and derivatives hold great promise for cancer prevention and treatment [3-5]. It is now believed that majority of cancers are attributed to environmental and dietary factors and incorporating fruits, vegetables, whole grains and spices in the diet seem to reduce the incidence of various cancers [6-9]. Several plant components exhibit superior anticancer properties by overcoming limitations of chemo- and targeted-therapies. Several plant compounds used in traditional medicine are common dietary ingredients and, hence, are considered safe [10]. Plant-derived anticancer agents are broad specific and affect multiple pathways simultaneously. Some plant constituents inhibit tumor-promoting pathways while also activating tumor-suppressor pathways [11,12]. Plant-derived agents have been effective at reducing inflammation and the level of reactive oxygen species (ROS), both common hallmarks of the genesis and progression of all cancers [13,14]. Certain plant components capable of killing cancer cells are also known to suppress the tumor promoting actions of immune and other tumor stromal cells [15]. In addition, plant products are less expensive and readily available. Intense research in this area has lead to multiple ongoing clinical trials involving plant derivatives while a few have already been approved for human use [16-18].

Fenugreek is commonly used as a traditional dietary ingredient in India and Egypt, among others. As a medicinal herb it has proven activity against hepatotoxicity [19], diabetes [20,21], hyperlipidemia [22] and cardiovascular diseases [19,23,24]. Fenugreek has anticancer properties [25] and has proven to be effective in preventing colon [26] and breast cancers [27]. Fenugreek extract also showed stimulatory effect on immune functions of mice [28].

More research on underlying molecular mechanism of the anticancer activity of fenugreek may lead to its development as an effective therapeutic/chemopreventive agent against various cancers. In this *in vitro* study we show, for the first time, that fenugreek causes death of T-lymphoma Jurkat cells by inducing autophagy.

### Materials

The cell culture medium (RPMI-1640), fetal bovine serum (FBS) and penicillin-streptomycin were purchased from Gibco-BRL Life Technologies Inc. Jurkat cell line was obtained from American Type Culture Collection (ATCC), USA. Dry seeds of fenugreek, fennel, black pepper, coriander and cumin and sticks of cinnamon used in this study were of food grade and obtained from commercial sellers in Riyadh.

## Methods

### Preparation of spice extracts

1.5 g of each of the finely ground spices was suspended in 50% ethanol in tightly capped bottles and shaken overnight in water bath maintained at 40°C. The spice suspensions were filtered and the filtrates evaporated under N_2_ and the dry residues resuspended in 1.5 mL of 50% ethanol to obtain stock solutions of 1 g/1 mL. Further dilutions of fenugreek extract were made by mixing with RPMI medium. Stock solution of fenugreek extract was directly used for GC-MS.

### Cell culture

The Jurkat cell line was cultured in RPMI-1640 medium supplemented with FBS (10%, v/v), streptomycin (100 μg/mL) and penicillin (100 U/mL). 5 X 10^4^ cells/mL were distributed into 24 well plates (1 mL/well) and incubated under 5% CO_2_ in a humidified atmosphere at 37°C.

### Cell viability assay

Cell numbers and viabilities were assessed using a hemocytometer based on the ability of the viable cells to exclude trypan blue. Briefly, at the end of treatment period cells in the wells were mixed well and an aliquot of cells were mixed with an equal volume of 0.4% trypan blue and after 2-3 minutes viable cells were counted by hemocytometer. Viable cells were expressed as a percentage of cells in the untreated well, which at the end of the incubation period was considered 100%. Cell numbers with standard error were averaged from 3 independent experiments.

### Morphological evaluation of cells

Normal and fenugreek extract treated cells were photographed using inverted light microscope at a magnification of 400x.

### Quantification of autophagy associated genes expression by RT-PCR

Total RNA was isolated from Jurkat cells using Qiagen RNeasy mini kit (Qiagen). RNA was reverse transcribed into cDNA using QuantiTect Reverse Transcription Kit (Qiagen). qRT-PCR was performed using SYBR Green PCR kit (Qiagen). Primers used for RT-PCR are shown in Additional file 1: Table S1. The master mixes were pipetted into a 96-well plate followed by the addition of 40 ng of RNA. All samples were analyzed in triplicate. PCR was run using the Bio-Rad Real-Time PCR System which was programmed as follows: (1) 95°C stage for 10 minutes, and (3) 40 cycles alternating between 95°C for 15 seconds and 60°C for 1 minute. Results were analyzed by comparative Ct method using the formula: 2^-ΔΔCT^. ΔC_T_= C_T_ value of gene of interest minus C_T_ value of β-actin. The ΔΔC_T_ was calculated by subtracting the ΔC_T_ of the untreated cells from the ΔC_T_ of the test sample.

### Gas chromatography–Mass spectrometry (GC-MS) analysis

GC/MS analysis of fenugreek extract was performed using an Agilent 7890 GC System Gas Chromatograph interfaced to a Mass Spectrometer (GC/MS) equipped with a VF-5ms capillary column (30 m × 0.25 mm ID × 0.25 μm). GC/MS detection was achieved using an electron ionization system with 70 eV ionization energy. Helium (99.999%) was used as carrier gas at a constant flow rate of 1 mL/min. An injection volume of 2 μL was employed in splitless mode. Injector and ion-source were maintained at 250°C and 280°C, respectively. The oven temperature was raised from 100°C (isothermal for 2 min.) to 200°C at a rate of 5°C/min and further up to 240°C by 10°C/min and maintained at 240°C for 11 min. Total GC running time was 40 minutes. The relative amount of each constituent was calculated by measuring corresponding peak area and represented as a percentage of the sum of areas of all peaks. MS Workstation 7.0 software was used to analyze mass spectra and chromatograms.

### Statistical analysis

Data was analyzed using the Microsoft Excel (Microsoft Office 2007). Variables were presented as percentages (%).

## Results

### Screening spices for cytotoxicity against Jurkat cells

**Figure 1:**
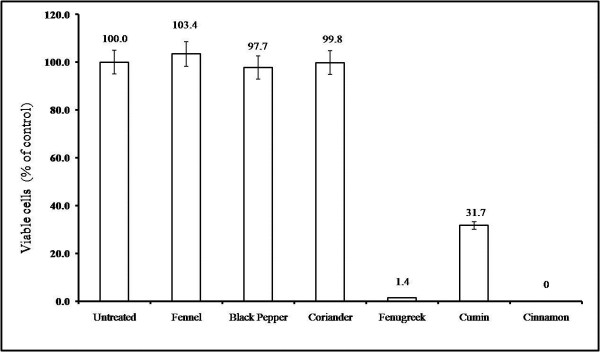
**Effect of different spice extracts on viability of Jurkat cells.** Jurkat cells were incubated with 1mg/mL of 50% ethanolic extracts of different spices for 72 hr. Viable cells were counted by trypan blue assay as described in methods. Fenugreek and cinnamon extracts were highly cytotoxic and affected the viability of most of the cells.

Jurkat cells were incubated with 50% ethanolic extracts of 6 different spices at 1 mg/mL for 72 hr and viable cells counted at the end by trypan blue exclusion assay (Figure 1). Cinnamon and fenugreek treatment resulted in death of 100% and 99% of cells, respectively. Only 25% viable cells were found after cumin treatment. Ginger, black pepper and fennel extracts showed no effect on the viability of Jurkat cells. Due to the distinct morphological changes induced by fenugreek extract it was chosen for further studies.

### Effect of fenugreek extract on the viability of Jurkat cells

**Figure 2:**
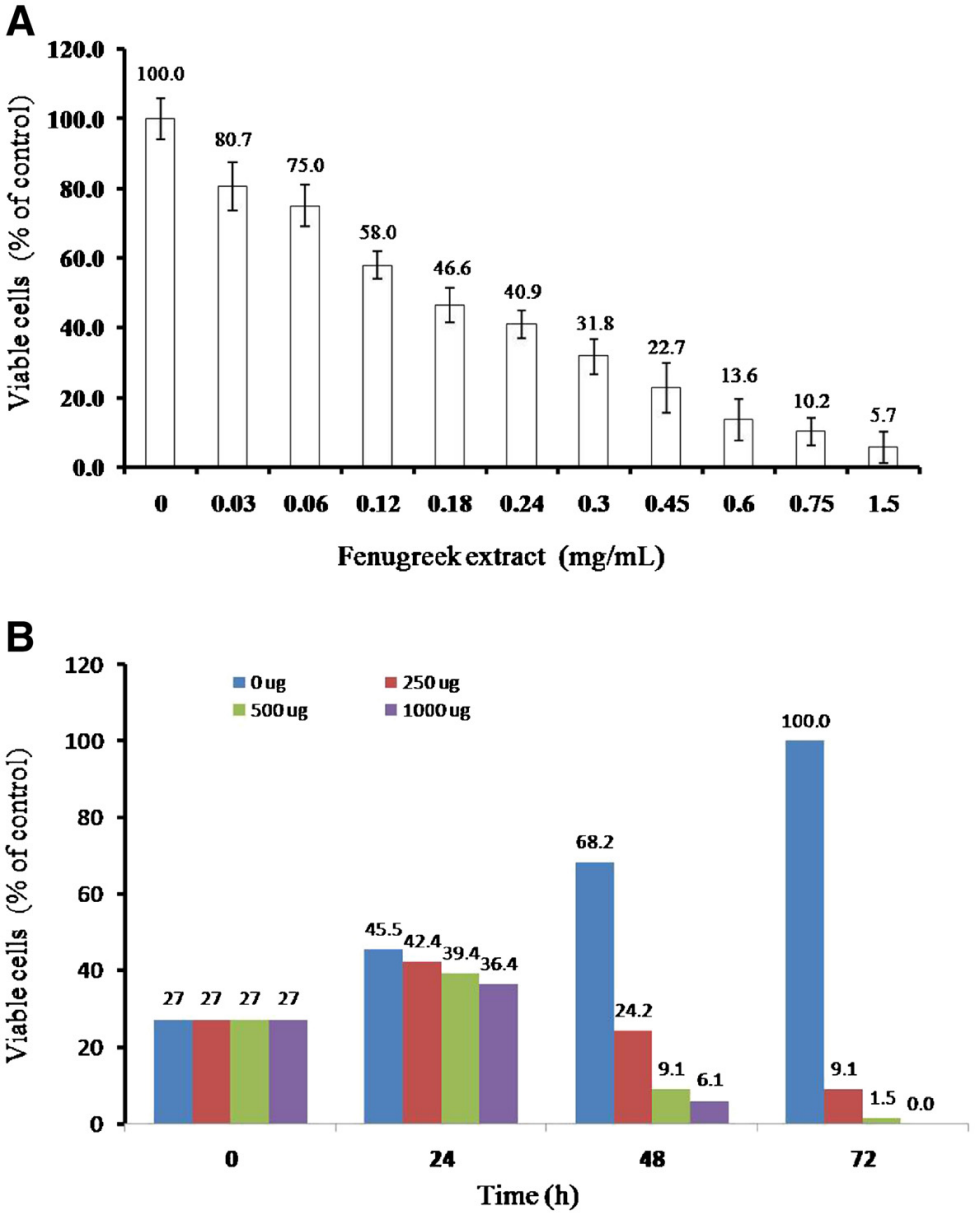
**Fenugreek extract inhibits Jurkat cell proliferation.** (**A**) Viability of Jurkat cells incubated with 30 to 1500 μg/mL of fenugreek extract was determined after 48 hr by trypan blue assay. Death of Jurkat cells occurred at all the tested concentrations of fenugreek and followed a dose-dependent pattern. (**B**) Time-dependent increase in death of Jurkat cells upon incubation with various concentrations of fenugreek.

Jurkat cells were incubated with 30 to 1500 μg/mL of fenugreek extract for 48 hr and viable cells counted at the end. Percentage of viable cells showed a steady decrease with increasing concentrations of fenugreek (Figure 2A). Cell death was observed even at 30 μg/mL and most of the cells were lost at 1500 μg/mL. Jurkat cells incubated with 250, 500 and 1000 μg/mL concentrations of fenugreek extract for up to 72 hr indicated dose and time dependent decrease in viability (Figure 2B). In this period, untreated cells almost doubled in number in a day for 2 days and continued to grow significantly on the 3^rd^ day. At all the three concentrations of fenugreek most of the cells were lost at 72 hr. Decrease in viable cells caused by fenugreek extract was not matched by a corresponding increase in dead cells by the trypan blue assay and this may be due to the complete disintegration of plasma membrane as shown in the following sections.

### Morphological changes induced by fenugreek in Jurkat cells

**Figure 3:**
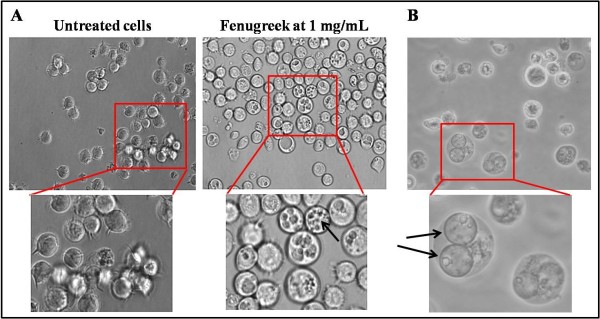
**Fenugreek extract induced vacuoles in Jurkat cells.** Overnight incubation of Jurkat cells with 1 mg/mL of fenugreek extract induced distinct vacuoles. (**A**) The number of vacuoles range from 1 to several per cell. Arrow points to multiple vacuoles in a cell. (**B**) Arrows point to Jurkat cells possessing two large spherical vacuoles. Pictures represent inverted light microscopic images taken at 400x magnification.

**Figure 4:**
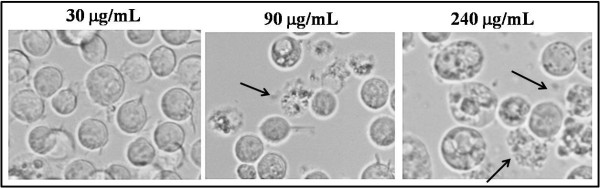
**Fenugreek extract induced plasma membrane rupture and total cell lysis.** Increasing concentrations of fenugreek extract after 48 hr caused morphological changes leading to cell death. Disappearance of cell shape by rupture of plasma membrane was seen at higher concentrations of fenugreek extract. Arrows point to cellular membranes undergoing rupture. Pictures represent inverted light microscopic images taken at 400x magnification.

Incubation of Jurkat cells with fenugreek extract resulted in the appearance of vacuoles at all concentrations of fenugreek tested (Figure 3A). These vacuoles were large enough to be seen directly by light microscope. Vacuoles appeared from as early as 16 hr of incubation of cells with higher concentrations of fenugreek (>250 μg/mL). Some cells had a single large vacuole occupying most of the cell space and others had more. The vacuoles appeared highly spherical (Figure 3B). Appearance of multiple large vacuoles was followed by disintegration of plasma membrane (Figure 4). With increasing concentrations of extract, some of the cellular contents could be seen in the absence of distinct plasma membrane and even these cytoplasmic cell structures disappeared over time, indicating complete cell lysis. Whole cell lysis was also indicated by the absence of dead cells upon staining with trypan blue even through the number of viable cells showed large declines.

### Fenugreek extract induced up-regulation of LC3

**Figure 5:**
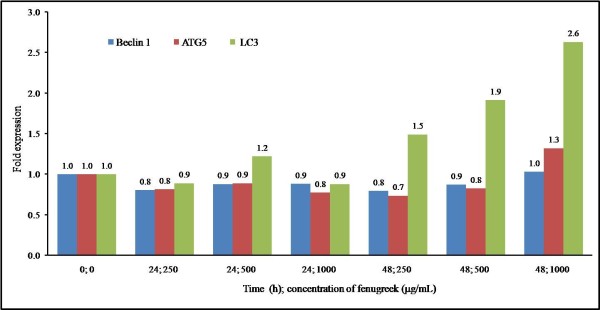
**Transcriptional regulation of autophagic genes by fenugreek.** Jurkat cells incubated with 250, 500 and 1000 μg/mL of fenugreek extract were quantitatively analyzed for transcripts of autophagic pathway genes beclin 1, ATG5 and LC3 by RT-PCR using β-actin as internal reference. Dose dependent increase in LC3 transcripts was found in cells incubated for 48 hr while no significant changes were observed for beclin 1 and ATG5.

Jurkat cells were incubated with 250, 500 and 1000 μg/mL concentrations of fenugreek extract for 24 and 48 hr. RT-PCR analysis of RNA from these cells indicated up-regulation of LC3 relative to β-actin in samples harvested at 48 hr and the increase depended on the concentration of fenugreek (Figure 5). A 2.5 fold increase in LC3 transcript was found in cells treated with 1000 μg/mL of fenugreek compared to untreated cells. However, the quantity of LC3 transcripts did not differ much from the control at the end of 24 hr.

### GC-MS analysis of fenugreek extract

GC-MS analysis of fenugreek extract resolved into 34 peaks corresponding to a similar number of unique compounds. Gingerol (4.82% of the total), cedrene (2.91%), zingerone (16.5%), vanillin (1.52%), β-bisabolene (1.25%) and eugenol (1.25%) were some of the anti-cancer compounds identified by GC-MS in this extract (Table 1).

**Table 1:** Anticancer compounds of 50% ethanolic extract of fenugreek seeds identified by GC-MS

**Retention Time (min.)**	**Compound Name**	**Formula**	**% of Total**	*Activity
8.54	4-Vinylguaiacol	C9H10O2	1.36	Anti-edemic; anti-inflammatory; anti-prostaglandin; prostaglandin-synthesis-inhibitor
9.452	Eugenol	C10H12O2	1.25	Antibacterial; anti-inflammatory; antioxidant; antioxidant; antitumor; cancer-preventive
10.463	Vanillin	C8H8O3	1.52	Anticancer; antimutagenic; antioxidant; antitumor; antitumor-promoter; antiviral; cancer-preventive; fungicide; immunosuppressant
13.05	β-Bisabolene	C15H24	1.25	Anticancer
13.488	Cedrene	C15H24	2.91	Cancer-preventive
16.486	Zingerone	C11H14O3	12.03	Anti-inflammatory, antioxidant
29.508	Gingerol	C17H26O4	4.82	Antiemetic; anti-hepatotoxic; antihistaminic; anti-inflammatory; antioxidant; antiseptic; anti-thromboxane; cancer-preventive; cyclooxygenase-inhibitor; hepatoprotective

*Source of anticancer functions of fenugreek constituents: Dr. Duke's phytochemical and ethnobotanical databases (online database).

## Discussion

Plant derivatives have great promise as anticancer agents. In this study, low concentrations of fenugreek extract was found to induce death of Jurkat cells, a commonly used cell line model to understand anticancer functions and underlying mechanisms of exploratory drugs or compounds. Fenugreek extract affected the viability of Jurkat cells in a dose- and time-dependent manner. Fenugreek extract-induced death was preceded by dramatic morphological changes involving the appearance of multiple large vacuoles and transcriptional up-regulation of LC3, both of which imply activation of autophagy. Results from this study imply autophagy as a novel mechanism by which fenugreek may bring about death of preneoplastic/neoplastic cells. T-cell lymphoid malignancies are a group of highly aggressive diseases that are generally resistant to current treatment modalities and results from this study indicate benefits of fenugreek as a potential therapeutic. Further, induction of autophagy may be a general anticancer mechanism underlying chemopreventive/therapeutic function of fenugreek.

Anticancer properties of fenugreek or its constituents involve multiple functional and molecular targets. Fenugreek induced apoptosis in a wide variety of tumor cell lines, including those of human colon [26], osteosarcoma [29], leukemia [30], breast [31,32], and liver [33]. Fenugreek blocked migration and invasion by reducing matrix metalloproteinase expression in human prostate cancer PC-3 cells [34], decreased nitric oxide (NO) and prostaglandin production by suppressing iNOS and COX-2, respectively, in an osteosarcoma cell line [35]. Fenugreek blocked activation of NF-KB, I kappa B kinase and AKT and suppressed the production of various proinflammatory cytokines like IL-6, IL-1 and TNF-α by cancer cells [31,36]. An earlier study noted the formation of cytoplasmic vacuoles in breast tumor tissue of 7, 12-dimethylbenz (α) anthracene (DBMA) treated rats upon feeding of fenugreek compared to control [27]. To our knowledge, ours is the first report to show death of human cancer cells by fenugreek-induced autophagy.

Apoptosis and autophagy are important pathways designed to cause programmed death of stressed cells and cancers suppress both these pathways and evade death. Autophagy has been defined as a process of transport of cytoplasmic contents to the lysozomes in double membrane compartments, called vacuoles [37]. Autophagy is a physiological response to stress and has been suggested to enable cells to adapt and survive and, hence, is considered a pro-survival mechanism [38]. Autophagy has also been defined as a highly conserved process of programmed cell death [39] and its activation causes cell death [40]. Defective regulation of autophagy in cancers suggests that autophagy is a true tumor-suppressor pathway [41,42] and this is further supported by the fact that several commonly activated oncogenes (for example, those encoding PI3K, TOR, Bcl-2) inhibit autophagy, whereas commonly mutated or epigenetically silenced tumor suppressor genes (such as those encoding p53, PTEN, TSC1/TSC2) stimulate autophagy [43].

Several studies have used electron microscopic methods to detect autophagic vacuoles. However, fenugreek-induced vacuoles in Jurkat cells were large and distinct and could be directly seen using an inverted light microscope. This study established the vacuolization as an autophagic process from the transcriptional up-regulation LC3. LC3 plays a critical role in autophagy and is considered suitable marker of this process [44]. In the present study, fenugreek extract increased LC3 transcript level with increasing concentrations of fenugreek extract over a period of 48 h. Harmol, a beta carotenoid, induced death of lung cancer cells A549 was associated with increased expression of LC3 [45] and siRNA knockdown of LC3 resulted in blockade of cell death, which, in turn, served to prove that harmol-induced autophagy was only a death mechanism [45]. Transcripts of beclin 1 and ATG5, the other autophagic pathway genes, did not show any decline in our fenugreek-treated Jurkat cells and this was similar to harmol-treated A549 cells which failed to show any changes in the expression and phosphorylation of beclin 1 [45].

Some health benefits of various medicinal plants or their constituents have been attributed to their ability to induce autophagy in diseased cells [46]. Experiments performed to measure the autophagy-inducing ability of several plant constituents, for eg., fisetin (found in tomatoes, apples, onions, and grapes), genistein (soybean) and quercetin (apple skins and red onions), report opposing findings (induction or suppression) resulting in reduced clarity on the beneficial effect of autophagy in cancer treatment. These contradictory results have been attributed to several factors, such as different methods employed to measure autophagy and the use of different cell types, such as cancerous cells versus normal cells [46]. Also, autophagy, at low levels, was suggested to serve a pro-survival role in tumors [47]. However, results from our study revealed increased production of autophagic markers and decreased viability to be associated with increasing concentrations of fenugreek implying only an anti-proliferative /pro-death function for autophagy at all concentrations of fenugreek extract. Results from our study support the notion that fenugreek-induced autophagy in Jurkat cells could only be an antitumor function.

Some of the components of fenugreek extract identified by GC-MS in our study have already been shown to possess significant anticancer activities and these include gingerol [14], cedrene [48], zingerone [49], vanillin [50], and eugenol [51-53]. Future studies will be designed to identify the specific compound(s) responsible for induction autophagy.

The cytotoxic effects of fenugreek are limited, mostly, to cancer cells [25,32]. Bioavailability of therapeutic components of fenugreek, unlike curcumin, is high as seen from its hepatoprotective effect from various in vivo studies [54,55]. Feeding studies have indicated tolerance and absence of any toxicities of up to several grams of fenugreek per day over several weeks [56].

The authors acknowledge some limitations. The study included a limited type and number of cell lines and lacked specific assays to confirm apoptosis and autophagy. Nevertheless, the present study identified autophagy as a new mechanism by which fenugreek may exert its therapeutic/chemoprevention/anticancer function.

## Conclusion

Fenugreek induced autophagy in Jurkat cells in a time and dose dependent manner, as indicated by morphological changes and up-regulated LC3. GC-MS analysis of the fenugreek extract identified several compounds having anticancer properties. Thus, due to its ability induce death of Jurkat cells by autophagy at low concentrations and lower level of non-specific toxicity, fenugreek may serve as a potential therapeutic in the treatment of lymphoid malignancy.

## Competing interests

The authors have no competing interests to declare.

## Authors’ contributions

SK and OEA performed cell culture experiments. AKM conducted RT-PCR quantifications. SHA and SMY were involved in preparation of spice extracts and GC-MS analysis. SK, MSA and KMA designed the experiments and helped to draft the manuscript. NMD conceived and coordinated the study. All the authors have read and approved the final manuscript.

## Pre-publication history

The pre-publication history for this paper can be accessed here:

http://www.biomedcentral.com/1472-6882/12/202/prepub

## Supplementary Material

Additional file 1 **Table S1.** Primers used for analysis of expression of autophagy associated genes by RT-PCR. (DOC 160 kb) Click here for file
