# Supplementary material for: Fenugreek extract as an inducer of cellular death via autophagy in human T lymphoma Jurkat cells
Source: BMC Complement Altern Med. 2012 Oct 30;12:202. doi: 10.1186/1472-6882-12-202 (PMC3520713; doi:10.1186/1472-6882-12-202)
Supplement: Additional file 1 — Table S1. Primers used for analysis of expression of autophagy associated genes by RT-PCR. (DOC 160 kb) [file 1472-6882-12-202-S1.doc]

**Supplementary Table 1. Primers used for analysis of expression of autophagy associated genes by RT-PCR**

| **Gene Forward Primer (5′-3′) Reverse Primer (5′-3′)** |
| --- |
| Beclin 1 AGGAACTCACAGCTCCATTAC AATGGCTCCTCTCCTGAGTT  ATG5 CACAAGCAACTCTGGATGGGATTG GCCACAGGACGAAACAGCTTC  LC3 CCGTCGGAGAAGACCTTCAAGC GGTTGGATGCTGCTCTCGAATAAG  β-actin TCATGAAGTGTGACGTTGACATCCGT CCTAGAAGCATTTGCGGTGCACGATG |
